# ‘Beyond’ Human Enhancement — Taking the Developing Country’s Perspective Seriously

**DOI:** 10.1007/s41649-021-00193-z

**Published:** 2021-10-08

**Authors:** Vorathep Sachdev

**Affiliations:** The University of Edinburgh, Edinburgh, UK

**Keywords:** Human enhancement, Buchanan, Development, Neoliberalism, Medical tourism

## Abstract

Bioethicists and philosophers dominate the on-going debate on human enhancement. They have debated the definition of human enhancement as well as the potential impacts of human enhancement technologies (such as pharmaceutical enhancements or pre-natal selection). These discussions have percolated, through bioethics bodies and bioethics recommendations, policy makers and have eventually been translated into policy. While some suggestions have been based largely in Western liberal democracies, others have deliberated the geopolitical consequences of human enhancement technologies. This paper argues that the present debate currently lacks perspectives from developing countries. It begins by introducing the current debate on human enhancement and recognizes Allen Buchanan’s well-raised concerns on how these technologies may potentially cause new injustices for low- and middle-income countries (‘developing countries’). It then provides two arguments calling for further research into human enhancement from the perspective of developing countries. First, this paper will argue that the current frames with which enhancement technologies are viewed are inherently neoliberal and require change. The second argument shows how the potential impacts of human enhancement technologies in developing countries have not been fully realized by analyzing how human enhancement technologies will impact Thailand, a developing country.

## Introduction

There is significant evidence that bioethical recommendations are considered by policymakers while creating policy (Cabrera 2015), making the deliberations of bioethicists important for the general public and the world’s geopolitical climate. Human enhancement (‘enhancement’) and human enhancement technologies (‘enhancement technologies’) have been subject to great debate in the bioethical arena, what was merely speculative and science fiction at first is now almost reality (Quigley and Ayihongbe 2018). While the definition of enhancement is by far an unsettled one, this paper does not intend to debate several meanings of enhancement. As such, it adopts the Welfarist account of enhancement that is almost synonymous with the dictionary definition of the word ‘enhancement’: “an intervention which increases the chance of a person having a good life” (Savulescu 2006). Setting out a simple definition allows us to move past debates on the distinction between enhancement and therapy and focus on bioethical debates regarding the possible consequences of enhancement, and enhancement technologies (means or interventions by which enhancement is achieved) (Buchanan 2011). Enhancement technologies include cognition-enhancing pharmaceuticals like Adderall or more invasive interventions such as the genetic modification of embryos (Ricci 2020).

These enhancement technologies have the potential to impact the whole world (Bess 2007), yet much of the debate has been dominated by bioethicists and has primarily been conducted in the West, both the positive spin on enhancement as well as the egalitarian concerns have primarily been based in Western liberal democracies (Hogle 2005). In 2005, anthropologist and bioethicist Linda Hogle recognized the insufficient focus on developing societies and political consequences of enhancement in bioethical literature when she stated:

Yet the overriding bioethics focus on Western notions of fairness and equity, risk, and prescriptive judgments for policy purposes excludes analysis of social disparities, differences in local political, economic, and health conditions, and differing value systems that are central to anthropological understandings of health and medicine. What might enhancement mean in a poor society where an artificial limb specially designed for working in rice fields or a bicycle designed to provide mobility means the difference in a person’s ability to make a living?

Following this, the debate remained largely as a Western phenomenon (Morrison 2015) and when it has addressed the impact that these technologies may have on developing countries, for instance in Allen Buchanan’s book, ‘Beyond Humanity’ it has misunderstood these countries. Thus, this paper argues that these misunderstandings be corrected and calls for more research that suitably represents the perspectives of developing countries through two key arguments.

Argument 1 puts forth that the current framework and mindset used to study human enhancement in relation to developing countries needs to be reconsidered. It challenges the neoliberal nature of how human enhancement is currently viewed and then requires that the cultural and political notions shaping developing countries to be the starting point with which we view human enhancement for the purpose of developing countries. Philosopher Julian Baggini (2018) aptly put why it is important to do so by stating:

If we forget when and where they wrote [non-Western philosophers], we are doomed to misunderstand them. But if we fail to see how what they say applies to here and now, we are doomed to waste or misuse them.

Argument 2, on the other hand, posits that there are several unconsidered consequences that may occur from the application of enhancement technologies in developing countries. By using Thailand as a case study, it analyzes how *one* such unintended consequence is human enhancement tourism, and concludes that the potential impacts of human enhancement technologies will differ considerably from each other. Such unintended consequences are yet to be realized in the current literature.

Notably, this paper should be viewed as a nyumon. A nyumon is a Japanese term for defining a physical space but also for inviting visitors in, Japanese writers translate it to the word ‘introduction’, where one is not told everything about another but given the opportunity to learn more about them and begin an acquaintanceship (Baggini 2018). Similarly, this paper invites the reader to understand the possible frames with which enhancement technologies should be considered and the possible consequences they may have on a developing country while still leaving the opportunity for more research to be conducted in the hopes that a fruitful acquaintanceship may begin.

## State of the (Inadequate) Debate

While this paper will not expound upon all the important arguments raised along the bioethical spectrum, it provides a brief introduction to the enhancement debate here. It is an attempt to engage with the arguments (or lack thereof) related to developing countries and briefly explain the main arguments engaged with or critiqued in this paper.

There are both permissive and restrictive views on enhancement, with those in favour of enhancement known as bio-liberals and those against known as bio-conservatives (Giubilini and Sanyal 2015). Bio-liberals, or the permissive end of the enhancement spectrum, do not object to human enhancement. Some go as far as calling it morally obligatory, who believe that the future is in enhancements: this group are better known as transhumanists (Bostrom and Sandberg 2009). For example, Julian Savulescu posits that parents should “select the child, of the possible children they could have, who is expected to have the best life, or at least as good a life as the others, based on the relevant available information” (Savulescu 2001). Nick Bostrom argues that pharmaceutical companies should focus on creating cognitive enhancement drugs, as a more intelligent society will eventually lead to a more equal Western liberal society (Bostrom and Sandberg 2009).

On the other hand, bio-conservatives have principle objections and practical objections to enhancement. The principle objections range from theological objections such as ‘playing god’, to fear that enhancement will change what it means to be truly human, for instance, will the moral worth of being human be different to what it will be if there are enhanced humans? Will enhanced humans be morally valued more than that of an unenhanced human (Peters 2012; Giubilini and Sanyal 2015)? Alternatively, some are afraid that this would infringe on the liberty of future generations who have been enhanced without their consent, for instance in pre-natal selection scenarios. In contrast, practical objections are concerned with the unintended consequences that can come from playing with the complexity of human design, analogous to how the environment has been unintentionally destroyed in the past (Healey and Rayner 2009). Further, philosophers fear a ‘liberal eugenics’, unlike the previous state-led eugenic programs, the fear is that individuals may choose to impress their own biases (for instance racial or gender biases) and further oppress minorities in Western liberal democracies (Agar 2004). Similar egalitarian concerns are related to the slow diffusion of enhancement technologies, potentially creating generational inequalities or further increase the gap between the wealthy and the poor in a Western liberal democracy (Giubilini and Sanyal 2015).

However, some philosophers, such as Allen Buchanan, have raised concerns regarding the impact that these technologies may have on low- and middle- income countries (‘developing countries’). Given its global impact, he is concerned with new injustices that may possibly arise from the slow diffusion of enhancement technologies (Buchanan 2011). While he has adopted an uncommon but positive approach in looking at human enhancement from this geopolitical perspective, his arguments can and should be refined to represent the perspective of a developing country more accurately, as this paper will posit.

Buchanan takes the stance that enhancements are inevitable — there’s no stopping their development. He first presents an argument in favour of enhancement using a historical perspective. This is an example of how he portrays enhancement as positive and part of our history of improvement — it is significant in this paper’s forthcoming critique of the enhancement debate’s and Buchanan’s neoliberal views. He compares biomedical enhancement to that of education, where education played a crucial role in increasing our cognitive abilities and thereby increasing productivity. While he is aware that productivity does not equal better well-being, he emphasizes that history has shown there to be a positive correlation. For example, agricultural technologies allowed people to group together and live in one area with enough food supply, and eventually allowed more individuals to focus on ‘mental’ work. Crucially, he has painted a picture of improvement and promise, which he then connects to the theory of development (Buchanan 2011).

Assuredly, he recognizes that taking the lens of development for this issue “can encourage a greater appreciation for the complexity of the issues of distributive justice that enhancement raises” (Buchanan 2011). Allen Buchanan’s key premise is that the developing world suffers injustices from the inadequate diffusion of highly valuable technologies. He spots the geopolitical differences between the developed world and developing world, addressing those new injustices may be created from the slow of diffusion of technologies. He states that the slow diffusion of enhancement technologies in developing countries may give “unacceptable advantages to those in political power to those who do have access or by excluding those who lack access to them from important sites or forms of economic cooperation” (Buchanan 2011). From this, he means both geopolitical considerations such as a state in political power that can take advantage of another state in exchange for providing enhancement technologies. Or more organic forms of advantage, take enhanced humans who may receive employment in countries where access to enhancement technologies have not yet been provided, making those in that country worse off than they initially were.

Buchanan believes that should the diffusion problem be solved; enhancements will help improve and alleviate inequalities in developing countries. For example, vaccine delivery programs in developing countries have significantly reduced child mortality rates. As such he proposes an institutional solution, namely the GIJI or Global Institute for Justice in Innovation, that resembles the WTO (World Trade Organization) in how it is created and how it holds authority, that is to say it will be a result of a multilateral treaty that can make decisions that only affect international law (Buchanan 2011).

Unfortunately, the enhancement debate in relation to developing countries has not moved much forward in the past decade, rather Buchanan’s book has remained as the largest contribution yet. As a result, this paper essentially critiques Buchanan’s arguments while building its own arguments. Paulo and Bublitz (2019) reflect on a decade of the enhancement debate and the pro-enhancement arguments that claim that moral bioenhancements may be the solution to humankind’s largest issues, for example, David DeGrazia (2014) referring to genocides in Rwanda and Bosnia or Molly Crockett discussing political conflict. Critically, Paulo and Bublitz (2019) recognized that the current debate still lacks a “thorough analysis of the causes” and in turn “leads to an overemphasis of specific means”. Rather, this paper’s first argument suggests that we avoid “the reductionist framing of global problems as deficits” (Paulo and Bublitz 2019), by critiquing the neoliberal frame with which past arguments have been put forward and suggest a frame that may suit the developing country instead.

## Argument 1 — Misunderstood: Do We Truly Understand the Social Context of Developing Countries?

Julian Baggini puts it very well, “sometimes, simply by changing the frame, the whole picture can look very different” (Baggini 2018). This argument is directed at the overarching theme of the pro-enhancement debate that pushes the idea (or in this case, frame) that enhancements can be beneficial and alleviate global poverty and injustice. As noted above, several moral enhancement arguments have been put forward as a solution to solve global justice, yet the issue of contextual analysis remains (Paulo and Bublitz 2019). Merely two years ago, Oliver Feeney (2019) too called for a change in framing:

To this end, there is an urgent need for an alternative ‘default’ framing giving a greater role for sociological input (i.e. the pervasive effects of social structures) into the normative debates on genome editing, as particularly notable in the enhancement myth, for more balanced and productive discussions, attitudes and policy approaches.

Using Buchanan’s (2011) proposed institutional solution, the GIJI, this argument thus highlights the current neoliberal frame in the debate, how it misunderstands the context of a developing country and how this view must change (Sanyal 2016).

Generally, when the Third World — a symbolic name for countries that are categorized as developing — is included in policy considerations, concerns of poverty and technological divides are raised. New technologies, such as enhancement technologies, are used as tools of inspiration for solving these problems, legitimizing technology as the solution (Healey and Rayner 2009).

Joseph Weizenbaum put it perfectly (Oppenheimer 2003):

There is temptation to send in computers wherever there is a problem. There’s hunger in the Third World. So computerize. The schools are in trouble. So bring in the computers. The introduction of the computers, be it in medicine, education or whatever, is usually to create the impression that generous deficiencies are being corrected, that something is being done. But often its principled effort only serves to push problems further into obscurity — to avoid confrontation with the need for fundamentally critical thinking.

The GIJI illustrates how Buchanan recognizes the cause of new and old injustices to be the slow diffusion of innovative technologies as the problem and promises quicker distribution of innovation and enhancement to be the solution. He has obscured the problem further. His solution’s similarity to previous promises shall demonstrate this.

His solution’s, GIJI, goal will be to encourage research and innovation whilst promoting diffusion through entrepreneurs and NGOs. Its core component is the power to grant compulsory licenses, an outcome of his efforts to reduce the impact of intellectual property laws that allow producers to have monopoly-like powers that lead to higher royalty prices. Yet, the compulsory licensing mechanism would only be utilized as a last resort (ironically, delaying its intended goal of quick diffusion). The producers of the enhancement technologies would first be placed on a watchlist for slow diffusion, which would be followed by a public warning, before finally exercising the compulsory licensing mechanism as a final option. When the compulsory license is finally exercised, GIJI will fund a compensation scheme that is higher than what is required by current intellectual property laws using funds from membership fees, which includes a required membership fees provided by developing countries (Buchanan 2011).

Interestingly, the GIJI’s most potent power will be that of compulsory licensing. It is different to the current intellectual property regime (most member states falls under The Agreement on Trade-Related Aspects of Intellectual Property Rights — or TRIPs), where the power of granting compulsory licenses lies with the countries themselves (Cloatre and Pickersgill 2014). In doing so, developing countries have lost more control over this decision, had they actually had any in the first place. In India, TRIPs and the General Agreement on Tariffs and Trade allowed pharmaceutical companies to remove a compulsory license that India had placed initially (Al-Rodhan 2011). Similarly, the GIJI making the compulsory license a last resort will allow private companies to greatly benefit before any action even gets taken. Furthermore, when the compulsory license does get used, the royalty rates are higher than that of the royalty rate under TRIPs. Although the bill is footed by GIJI, GIJI is funded by its member states, where developing countries may have to take loans to pay their membership fees — further burdening them (Sanyal 2016).

According to Sanyal, the GIJI ignores the actual solution, which is political change to help global poverty (Sanyal 2016). He is not alone, as Thomas Pogge argues that former colonial powers have kept their power over former colonies through institutions such as the WTO. By allowing richer or developed countries greater protections through quotas, tariffs, subsidies to domestic producers and other similar mechanisms, developing countries have lost trillions of dollars in potential export revenue, not only leaving them stranded in poverty but forcing them to leverage their natural assets and governmental policies in exchange for loans from the World Bank. Pogge’s solution too is to change the institutions that currently exist today, but he takes a radical position that developed countries are reluctant to follow: the transference of wealth (Shapcott 2010). However, it is an example of the analysis that may arise when the perspective of a developing country is taken.

Even if we assume that the GIJI to be a useful solution, past lessons have made developing countries more aware of these false promises. The Green Revolution, successfully opposing the red revolution that promoted breaking up land holdings for the land-poor, was promised to reduce world hunger with new biotechnology allowing for higher-yield seeds (Sanyal 2016). In fact, at this point the Indian government had already learnt its lesson, decades earlier, a similar initiative begun with the production of genetically modified cotton. While genetically modified cotton dominated the Indian production, high fees left farmers and their families in debt, causing higher farmer suicide rates. They did not live, but their debts remained (Jasanoff 2016). The Supreme Court then initially declared a moratorium on genetically modified crops after learning its lesson, yet the Green Revolution left farmers hungry and again, in debt (Jasanoff 2016; Sanyal 2016). It is unlikely that after two similar incidents with biotechnology, the Indian government will repeat its mistakes.

This mistrust of new technologies is limited not only to India but has happened in the Philippines and other developing countries as well (Jasanoff 2016). Take Isa and Hj Safian Shuri’s (2018) analysis of human genetic enhancement in Malaysian Science Fiction Novels, where a mistrust of global privatization is rampant in one of its most famous human enhancement science fiction novels.

There is a problem in constantly looking at the developing country without understanding its notions or painting a world of promise. Vishwanathan, taking a stance against the views of promise and growth and technological solution proposed by American and European cultures, locates the South Asian perspective future technologies between the cosmological relations with God, man and nature and democracy, calling for greater democratic engagement, as the public holds values and critical ideas for innovation. He takes Third World futures, not as one of poverty but one of festival (Healey and Rayner 2009).

More importantly, Vishwanathan develops the South Asian perspective to emphasize the very idea that Argument 1 has tried to put forward, that:

The debates on life-enhancement and longevity belong to a similar cluster of discourses on progress, growth, development, and perfection. They look linear and morally innocent unless we confront their obverse side in the world of triage, obsolescence, waste, the defeated and the broken (Healey and Rayner 2009).

Allen Buchanan has taken strides in the bioethical debate on enhancement by even bringing development lens and framework in, however, since then the debate has been stagnant, but it cannot end here. The overarching ideas and discourses currently held regarding developing countries stills needs change. Cognitive Enhancement experts have also (Sattler and Singh 2016) highlighted this need through an example of the provision of cognitive enhancements (CE) to children within schools:

The provision of CE drugs to a group of healthy children (while it is still illegal for other children without a prescription) could easily lead to a kind of negative competition, in which parents with sufficient economic resources perceive drug provision to poor and disadvantaged children as a threat to their own children’s opportunities, with the consequence that advantaged parents put more energy into upholding their children’s privilege (with and without stimulants). Thus, educational settings could become battlegrounds for who does or does not meet criteria for sanctioned psychotropic CE. Societal attention to the structural inequalities that caused the problem in the first place could give way to lawsuits. Increased stimulant-demand could benefit pharmaceutical companies.

This example emphasizes how a change of frame brought forward a social context that was crucial in how the possible consequences were analyzed. Similarly, Argument 2 below continues to discuss how the possible consequences may differ in the context of developing countries.

## Argument 2 — How Enhancement Technologies Play Out in the Global South?

While the consequences to applied enhancement technologies are usually presented as solutions to poverty in developing countries or prohibited based on religious beliefs (Islamic bioethics for instance), in fact the consequences can vary completely. For instance, Malaysian perspectives on enhancement include survivalist form of enhancement, where enhancement technologies help countries at risk of being submerged in the water (Isa and Hj Safian Shuri 2018). On the other hand, the impact of enhancement technologies may not be as deterministic as assumed by the enhancement debate and especially Buchanan. This paper will take Thailand as a case study to explain how enhancement technologies may have different economic and social consequences in developing countries. This argument continues to act like a nyumon and aims to show that more research is required in the enhancement debate towards this, and that it will be beneficial to borrow from other disciplines such as anthropology, sociology, and STS.

STS literature has consistently provided a multidisciplinary view to subjects, and in this particular report, it discusses the politics of human enhancement from a European perspective (Jasanoff 2011; Coenen et al. 2014). This report considered various issues from a European political perspective, as it recognized that the broader societal, cultural, ethical and political frameworks have to be considered to reach a policy recommendation. The report considers ‘bottom-up tendencies in the politics of enhancement’, analyzing how enhancement technologies may attract market demand and impact policy. Take medical tourism, individuals traveling to other countries for interventions, may slowly include many enhancement interventions — creating an ‘enhancement tourism’, which the policy recognizes may impact distributive justice and health systems as well as medical risks (Coenen et al. 2014). It also identified that some forms of enhancement tourism already exist today, such as selecting between embryos after conducting pre-implantation genetic diagnosis (PGD) — an intervention used to help identify the genetic information of embryos (Coenen et al. 2014). Many developing countries are becoming hubs for medical tourism, Thailand is one of them, thus it makes it a good case study for the possible effects of existing enhancement tourism. It is also a good example because the possible impacts of enhancement tourism here can be extrapolated to developing countries in similar circumstances, for instance, Nepal, India or Mexico (Chuang et al. 2014).

Although medical tourism has existed for a long time, in the direction of developing countries to developed countries, the roles have recently reversed. Individuals from developed countries now travel to developing countries for medical interventions, usually due to lower costs or to circumvent the laws of their own country. For instance, Bumrungrad hospital in Thailand claimed to treat over 400,000 foreign patients in 2009 (Connell 2013).

When applied to enhancement, *both* positive and negative consequences may arise.

On the one hand, economic benefit, a promise Buchanan addresses, will attract foreign companies to invest in developing countries and help create the facilities required to facilitate medical interventions. In the case of surrogacy tourism in Thailand, a California-based organization set up multiple clinics in Bangkok and Phuket (a popular tourist destination in Thailand). As a positive, the creation of facilities will allow both foreigners and mid-level income locals to afford such technologies. Take IVF (in-vitro fertilization) tourism, progressing from 1 baby in 1987, IVF tourism, the birth point for inheritable enhancement technologies, has resulted in in over thirty clinics providing approximately 4000 IVF cycles in 2007 (Whittaker 2018). In addition, some public hospitals have also begun to provide IVF services in Thailand (Whittaker 2014).

On the other hand, medical tourism could lead to an uneven distribution of health resources, profit seeking companies are likely to make private businesses invest in technology-intensive medical interventions, for instance, enhancement interventions. It may seem positive from an enhancement viewpoint, however, this could cause legitimate employment and economic in the public health arena. Doctors may move to more wage-appealing private clinics from public hospital requiring public hospitals raising wages to prevent a massive internal brain drain, diverting money that would have initially been used directly for treatment. This did occur in Thailand, the budget for doctor’s wages have had a negative impact on resource allocation, in an effort to stop vacancies being created from doctor outflow to private hospitals. Over 6000 vacancies were created in 2005 alone, while private hospitals have grown by almost 30% (Chen and Flood 2013).

There may also be a cultural impact on developing countries such as Thailand. This can be distinguished into two categories, the cultural impact arising from international travel and the cultural impact on Thailand as a country resulting from how enhancement technologies are distributed due to enhancement tourism.

Medical tourism has helped to normalize interventions in developing countries. Again, in the case of surrogacy tourism, carrying another person’s child was initially considered a Buddhist sin but as time progressed, the term for surrogacy became known as “um-bun” — carrying for good (Whittaker 2018). Even in different culture and ideologies, such as feminism, medical tourism has helped normalize some attitudes. For instance, cosmetic enhancement was initially viewed by certain parts feminist scholarship as an oppression of patriarchal culture, however, over time this view changed to evolved to be “the motivation for cosmetic surgery as neither fully internal nor external but rather an inter-subjective and embodied process that takes place in a consumerist environment” (Whittaker 2018). While normalization can sometimes be seen as positive, impressing a Western view of how technologies are used in developing countries is another form of Western imperialism. Several studies found surrogacy tourism in Thailand and India to be representative of Western imperialism and colonialism (Lyzwinski 2013). White families flying in to have women of colour carry their child for them at significantly lower prices than they would pay in their home country, Donchin calls this a “post-industrial form of master-servant privilege.” In an enhancement tourism scenario, this form of cultural imperialism could occur if clinics only provided interventions that were appealing to white people, for example, enhancements to prevent ageing is primarily a Western idea, here the West represents nations who have a history as colonizers and are considered developed countries (Al-Rodhan 2011).

Developing countries, due to how colonization has played a role in the establishment of the nation state, have various cultures packed together into one country, sometimes causing cultural difference in different areas of the country. Consider Thailand, cultural differences between its northern regions and Bangkok have already established a class difference, causing some forms of discrimination (Whittaker 2004). These discriminations may be exacerbated, given the distribution of enhancement technologies in Thailand have been centralized to tourist centric cities such as Bangkok (Vutyavanich et al. 2011).

Even if the pre-existent attitudes did not exist in a developing country, the impact of enhancement tourism would likely be localized to common destinations, leaving behind many of the economically underprivileged areas in developing countries. A study by the Thai College of Obstetricians and Gynaecologists found that clinics for medical tourism were primarily located in the tourist centric parts of Thailand, such as Bangkok, this would leave most of Thailand bereft of enhancement technologies. This could possibly lead to inequality in ways that were dismissed by Nick Bostrom, in the context of Western liberal societies, and unforeseen by Buchanan when he discussed the possible practical implications of the diffusion of these technologies (Bostrom and Sandberg 2009; Buchanan 2011).

## NYUMON: A Stepping-Stone

This paper has tried to emphasize the idea that the content of the bioethical debate is not enough, context is equally, if not more, important. In bringing the context of geopolitical situations between developed and developing countries, Allen Buchanan started a very important debate in the bioethics arena, that becomes even more crucial when we consider that these debates may one day translate into policy. For example, the President’s Council on Bioethics (2003) prepared a report on human enhancement called ‘Beyond Therapy: Biotechnology and the Pursuit of Happiness’ for the American government. The report addressed many of the concerns mentioned in the previous part, such as questioning the meaning of being human, but also painted a picture of promise, a future that would be in line with the American dream. It is crucial for future policies to play out further geopolitical consequences and avoid this neoliberal policy framework.

This part attempts to thread together the above arguments with other arguments put forward in the bioethical debate on enhancement to demonstrate the need for more inclusive research into human enhancement using various perspectives such as STS.

Take Nick Bostrom, who promotes an argument for cognitive enhancement pharmaceuticals in order to improve society and reduce inequality. He advocates that higher intelligence will mean less social and economic misfortunes, and better health citing empirical evidence that one additional IQ point can raise a man’s income by 2.1% and a woman’s by 3.6%. Bostrom goes one step further and identifies a problem with the current framework, that drug companies struggle to receive regulatory approval to directly develop drugs for cognitive enhancement (Bostrom and Sandberg 2009). Cognitive enhancement drugs currently on the market were created to treat specific conditions but fortunately stumbled upon enhancing effects. He argues that legitimate progress towards equality can be made should these regulatory conditions change, and states provide cognitive enhancement pharmaceuticals to the public in a similar manner to education. While Bostrom notes that his argument is set in a Western liberal democratic society, enhancement technologies and pharmaceuticals have a worldwide impact (Bess 2007). Thus, if we situate his arguments in the context of a developing country, where pharmaceutical companies reap significant monetary advantages due to TRIPs, it is likely that large parts of the population will not receive access to these cognitive enhancement drugs, reviving the inequality debate as pointed out in Argument 1. For example, in Ghana — despite the encouragement of generic drugs, political play from pharmaceutical companies have allowed patented drugs to be more freely available and less affordable to the public. In Djibouti, where TRIPs does not play a large role, pharmaceutical advertisements, impractical donations from countries such as France, and French medical education have acted as silent forces in ensuring that pharmaceutical companies can sell at high, unaffordable prices (Cloatre 2013).

## Conclusion

This paper has emphasized how we will benefit from contextualizing the aims and values in locations other than Western liberal democracies and promising images of the future. It did so by first illustrating the imagination of promises held by current bioethical thinker and argued for viewing enhancements from different cultural attitudes. It then demonstrated one way in which contexts may change the consequences of how enhancements impact countries geopolitically and economically. Together, these arguments present how there is a need to explore bioethical arguments for the sake of future policy and the need for interdisciplinary approaches that can help construct new and legitimate arguments in the bioethics arena.
